# Assessing needs-based supply of physicians: a criteria-led methodological review of international studies in high-resource settings

**DOI:** 10.1186/s12913-023-09461-0

**Published:** 2023-05-31

**Authors:** Isabel Geiger, Laura Schang, Leonie Sundmacher

**Affiliations:** 1 Technical University of Munich, Munich, Germany; 2 Ludwig-Maximilians-University (LMU) Munich, Marchioninistrasse 15, 81377 Munich, Germany; 3 Pettenkofer School of Public Health, Munich, Germany; 4 Department of Health Economics, Technical University of Munich, Munich, Germany

**Keywords:** Need for healthcare, Physician capacity planning, Workforce planning

## Abstract

**Background:**

Many health systems embrace the normative principle that the supply of health services ought to be based on the need for healthcare. However, a theoretically grounded framework to operationalize needs-based supply of healthcare remains elusive. The aim of this paper is to critically assess current methodologies that quantify needs-based supply of physicians and identify potential gaps in approaches for physician planning. To this end, we propose a set of criteria for consideration when estimating needs-based supply.

**Methods:**

We conducted searches in three electronic bibliographic databases until March 2020 supplemented by targeted manual searches on national and international websites to identify studies in high-resource settings that quantify needs-based supply of physicians. Studies that exclusively focused on forecasting methods of physician supply, on inpatient care or on healthcare professionals other than physicians were excluded. Additionally, records that were not available in English or German were excluded to avoid translation errors. The results were synthesized using a framework of study characteristics in addition to the proposed criteria for estimating needs-based physician supply.

**Results:**

18 quantitative studies estimating population need for physicians were assessed against our criteria. No study met all criteria. Only six studies sought to examine the conceptual dependency between need, utilization and supply. Apart from extrapolations, simulation models were applied most frequently to estimate needs-based supply. 12 studies referred to the translation of need for services with respect to a physician’s productivity, while the rest adapted existing population-provider-ratios. Prospective models for estimating future care needs were largely based on demographic predictions rather than estimated trends in morbidity and new forms of care delivery.

**Conclusions:**

The methodological review shows distinct heterogeneity in the conceptual frameworks, validity of data basis and modeling approaches of current studies in high-resource settings on needs-based supply of physicians. To support future estimates of needs-based supply, this review provides a workable framework for policymakers in charge of health workforce capacity planning.

**Supplementary Information:**

The online version contains supplementary material available at 10.1186/s12913-023-09461-0.

## Background

Many publicly funded health systems embrace the normative principle that the supply and delivery of health services should be based on population need for care. As many countries struggle with regional inequalities in access to health professionals and questions about future surpluses or shortages of providers, respectively [1, 2], this issue is of high policy relevance internationally [2, 3]. However, while methods for forecasting future supply from estimated inflows and outflows have received much attention in the health workforce planning literature [4], the problem of population need for healthcare and how to translate it into an operational quantity for estimating needs-based supply remains elusive. This methodological study provides a framework for estimating needs-based supply of physicians and critically reviews international studies in high-resource settings, with results targeted specifically at health services researchers and policymakers in the field of health workforce planning.

Objective need for healthcare is a complex concept with two classical approaches that compete for its definition, namely: the humanitarian and the realistic theory [5]. According to humanitarian theory, there is a need for care when a person’s wellbeing is disturbed [6]. This theory focuses on the identification of diseases and human suffering [5]. Hence, the existence and extent of a need for care are equated with the level of ill-health in a population.

In contrast, proponents of the realistic theory argue that the recognition of a need for services is justified only when there is an effective intervention that improves the medical prognosis of a disease with some probability [5]. The basis of this argumentation is the methods and findings of evidence-based medicine. In addition, the realistic theory of need acknowledges the scarcity of financial, human, and technical resources available to society. Thus, there is oversupply if an additional unit of healthcare delivers little or no additional health benefit or if an existing potential benefit could have been met with a smaller amount of resources [7].

Therefore, Culyer [8] defines need for healthcare as “the minimum amount of resources required to exhaust a person’s capacity to benefit”. This definition requires, firstly, the availability of interventions likely to improve clinical outcomes (e.g. physical functioning) or quality of life (e.g. pain reduction, anxiety-relief). Thus, not every need for *health* entails a need for *healthcare* [7, 9, 10], when effective interventions are nonexistent or largely beyond the remit of healthcare systems [11, 12]. Secondly, an intervention cannot be said to be needed if another equally effective but less resource-intensive intervention exists [8, 13].

Following the realistic theory, the objective need for healthcare at a population level corresponds to the burden of morbidity that can be prevented or treated within the remit of health systems. Thus, objective need for healthcare can be considered a latent construct that cannot be observed empirically but must be approximated in relation to a theoretical approach [14]. The short systematization of the relationships among key determinants below is necessary for the subsequent, informed set-up of guiding criteria for estimating needs-based supply.

In our study, the objective need for health services is defined as avoidable or treatable morbidity that is amenable to care or prevention. It is further influenced by the current state of evidence-based medicine and the burden of morbidity in a population. The objective need correlates with exogenous determinants of morbidity such as demographic, environmental, socio-economic, and behavioral factors, which can in principle be recorded independently of actual service utilization and supply.

Demographic characteristics such as age and sex significantly influence the individual disease risk [15–20]. Individual health behavior such as diet, physical activity and smoking [21] also have a major impact on morbidity. The socioeconomic status of a person is clearly associated with the risk of disease and health behavior [22–25]. A person’s health is also determined by his or her social and material environment. Regional material deprivation (for example, as a result of noise and air pollution) can harm health directly but can also create psychosocial stress and influence health behavior with regard to diet [26], smoking [27], and physical activity [28]. Another social determinant influencing morbidity is the early life of a person, which was found to increase morbidity in later life [29, 30].

Additionally, endogenous elements of morbidity, which are part of the healthcare system, may also correlate with need for healthcare. Endogenous determinants affect the quantity and quality of service utilization [14], which in turn are affected by the available supply of services as well as the temporal and spatial accessibility. When existing supply fails to account for regional differences in need, this can fundamentally reinforce inequities in access to care if supply determinants are used as the main indicators for need [31–33]. Similarly, indicators of service utilization such as case volume or per capita expenditure may reflect some aspects of objective need for healthcare [34] but may deviate due to various factors including patient preferences, access to care and provider incentives, for instance with regard to supplier-induced demand [35]. Further ethical implications for the rationing and prioritization of resource allocation in healthcare as outlined by Brock and colleagues [36, 37] are beyond the scope of this paper as we define the objective need for healthcare independent from healthcare systems.

The third goal of the Sustainable Development Goals defines key targets to ensure healthy lives and foster well-being of all. One of its targets calls for universal health coverage including access to quality healthcare services [38]. Although countries in high-resource settings (like the United Kingdom (UK)) already offer universal health coverage, they fail to supply enough doctors to meet their population’s need for healthcare [39, 40]. Moreover, while physician supply was found to increase globally, national shortages across the world were found to persist, inhibiting high levels of universal health coverage [41]. Providing accurate estimates of the need for physicians can play a vital role to reduce current and avoid future shortages and excesses of physicians.

Thus, the overall objective of this review is to critically assess methodological approaches that estimate needs-based physician supply in high-resource settings using a set of guiding criteria based on central requirements. This criteria-led approach advances existing principles of workforce planning [4, 42, 43] by highlighting current gaps in the literature with a special focus on the relationship between morbidity, utilization, and supply. We aim to systematically identify how existing studies choose their conceptual framework, validate their data basis, select their model, translate need into physician capacity, and integrate future trends and developments in their predictions.

## Criteria for estimating needs-based supply

Based on the theoretical foundation of objective need and the practical requirements of health service planning, we set guiding criteria for the estimation of needs-based supply. The criteria were initially proposed in a scientific report to support/inform office-based physician planning in Germany [44] and include requirements for the conceptual basis, the data basis, the feasibility of the implementation for planning purposes, and the sustainability of the estimates of need (future changes and developments). As health system contexts and policy objectives differ, these criteria are meant to support the process of operationalizing needs-based supply, not to prescribe specific normative choices. Also, they do not raise claim of completeness. An overview of all criteria can be found in Table 1.

**Table 1:** Criteria for estimating needs-based supply of physicians and underlying questions

Criteria	Underlying questions
**1. Conceptual framework**	
1.1 Selection and justification of needs indicators	Is the selection of indicators theoretically well-founded and empirically supported for the respective context of the analysis?
1.2 Relationship between supply and need	Is the conceptual dependency of indicators of need on supply • in general, • regarding unmet need/lack of physicians or • regarding overuse/oversupply examined and, if possible, accounted for in the framework?
**2. Data basis**	
2.1 External validity	Is the population for which providers are to be planned and the population from which data are used identical or representative?
2.2 Internal validity	Does the observed data accurately measure the indicators of interest?
2.3 Timeliness and availability	Is the timeliness of data and availability of data sources reported, and considered with respect to the intended planning horizon?
**3. Modelling and translation into physician capacity**	
3.1 Transformation into provider requirements	Is the estimated need for healthcare related to some measure of provider productivity to transfer the estimated service requirement to physician capacities?
3.2 Model selection and validation	Is the statistical model appropriate and well-founded, and were the validity and the robustness of the findings established?
3.3 Level of analysis	Was the level of analysis defined and discussed regarding the potential for ecological errors?
**4. Integration of future trends and developments**	
4.1 Projection variables	Are projection variables identified that can be modelled according to future changes in population need for healthcare?
4.2 Planning horizon	Was the chosen planning horizon justified appropriately with respect to future changes?

### Criteria related to the conceptual basis

Since population need for healthcare cannot be measured directly, it must be operationalized using measurable indicators of treatable morbidity, hereafter called indicators of need. The motivation for specific indicators and their operationalization is based on different theories, and meaning of the indicators depends on the respective systemic and social context [45]. The influence of unemployment on morbidity, for example, interacts with the population’s access to health insurance policies and their specific benefit entitlements [46]. The selection of indicators should thus be theoretically well-founded and empirically supported for the respective context of analysis (Criterion 1.1). If no theoretical framework and empirical evidence is available, underlying assumptions should be stated.

A key challenge arises from the relationship between morbidity, utilization, and supply. In systems where healthcare providers accurately document a patient’s diagnoses (e.g. for the purpose of billing), these indicators can be used as determinants of (documented) morbidity. However, treatable morbidity may not only result in the use of medically indicated services, but also in the occurrence of both non-indicated services (“overuse”) and unmet need (“underuse”) [42]. A subset of diagnoses made by providers may therefore be related to non-indicated services while unmet need is, by definition, not documented. Unmet need and overuse can both result from the objective need for healthcare and from local levels of supply. Therefore, the conceptual dependency of indicators of need on supply should be examined and – if possible – accounted for in the model (Criterion 1.2).

### Criteria related to the data basis

Once suitable indicators of need have been identified, the data basis must be systematically evaluated. According to the criterion of external validity, the population for which providers are to be planned and the population from which data are used should ideally be identical or at least as similar as possible with respect to relevant indicators of need (Criterion 2.1). Therefore, empirical investigations with full coverage of the target population have a high degree of external validity. Other studies that are based on samples of the population, for example from cancer registries [47] or surveys of self-reported health status [48], should clearly state how far and with respect to which characteristics they are representative of the target population.

The internal validity of the data, in the sense that the observed data accurately measure what they are supposed to measure, should also be discussed (Criterion 2.2). Consideration should be given to quality and specifications for collecting data as well as to potential systematic biases and how they may be avoided. When indicators of morbidity are derived from the utilization of services such as documented diagnoses from billing data, one should reflect on whether the documented diagnoses consistently and appropriately map the “true” underlying morbidity, given potential biases due to the influence of healthcare supply on service delivery, potential unmet need and incentives related to comprehensive and accurate coding of diseases [49, 50].

Lastly, timeliness of data and availability of data sources should be considered with respect to the intended planning horizon [51]. This criterion also entails acknowledging potential limitations in comparability when combining several years of data and limitations that occur if the most recent data is unavailable (Criterion 2.3).

### Criteria related to the feasibility of the approach

Following the realistic theory of need for healthcare, estimating the burden of disease in a population is necessary but not sufficient for planning and implementation purposes. The extent of treatable morbidity must also be related to a required level of service and to the intensity of work to deliver these services [43]. The empirical quantity resulting from the operationalization of need should be related to some measure of provider productivity (Criterion 3.1), such as units of service per hour of work [42] or expected physician time required to care for different patient groups or specified levels of morbidity. The criterion allows a reasoned transfer of the estimated service requirement to physician capacities. For instance, estimates of need for services can be translated into provider requirements in terms of physician work hours and based on definitions of full time equivalents (FTE), which are often subject to changes over time [52]. Thus, each translation into physician capacities must identify the underlying assumptions and limitations.

While the specification of the model parameters, including the selection and quantification of the indicators of need, should be a well-founded decision, the selection of an appropriate statistical or analytical model depends on the characteristics of the data. Despite the fact that there are no universal criteria for model validation, different approaches such as the face, cross or predictive validation can be employed [53]. Additionally, it is important that the robustness of the findings is reasonably established through sensitivity analyses (Criterion 3.2).

If aggregated data is used to depict relationships that arise at the level of the individual, the relationships found at the aggregate level cannot necessarily be transferred to the relationships at the individual level. A variety of factors influence the relationship of variables at the aggregate level, and the generalization of the results could induce an ecological fallacy [44]. If relationships at the level of individuals are of central importance for the population needs assessment, they should be modelled at the appropriate level or critically discussed regarding the potential for ecological error [54] (Criterion 3.3).

### Criteria related to future changes and developments

Future changes in demography and epidemiology as well as the structure of healthcare provision may affect the selection of appropriate indicators of need, the data basis, and the modeling approach. Therefore, it is important to identify projection variables, which can be modelled according to theories on future changes in population need for services (Criterion 4.1). A closely related but distinct criterion is also the extent to which the chosen planning horizon has been justified with respect to these expected future changes [51] (Criterion 4.2).

## Methods

To highlight different approaches of operationalizing the need for healthcare and to identify potential gaps of published quantitative analyses of physician requirements, we conducted a criteria-led methodological review.

**Fig. 1:**
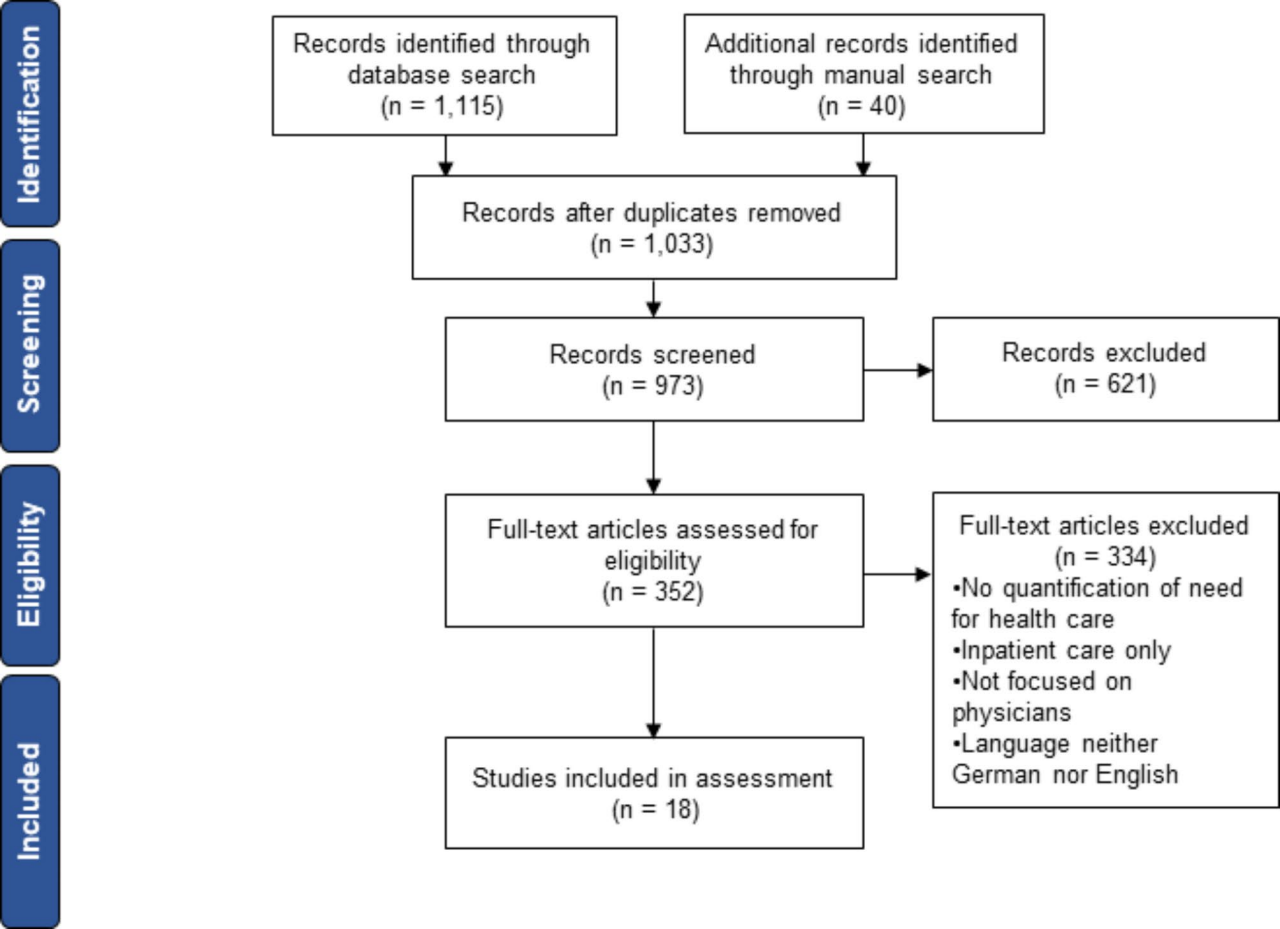
PRISMA flowchart based on Moher et al. [55]

The review was guided by the ‘preferred reporting items for systematic reviews and meta-analyses’ (PRISMA) framework by Moher et al. [55] and was synthesized in multiple steps (see Fig. 1). First, electronic bibliographic databases (PubMed, ScienceDirect and Web of Science Core Collection) were systematically explored for peer-reviewed articles that estimate needs-based physician supply using logical combinations of keywords (e.g. workforce planning, service requirement*, need). Results of the search were not limited using filters and Mendeley was applied as reference manager software [56]. A detailed list of all keywords and logical combinations per database can be found in Additional file 1. Second, manual target searches on national and international websites including (but not limited to) WHO Health Workforce, OECD and the EU Health Workforce Initiative to name but a few, were conducted to obtain relevant grey literature. Third, mining of references and author searches were employed to complement the findings. After identifying potentially relevant literature, initial screening was executed in a further step, starting with removing duplicates. Thereafter, abstracts or summaries were reviewed and screened before acquiring full texts. In a final step, predefined selection criteria were used to check eligibility (see Table 2). The review process was conducted by two independent reviewers. Disagreements were resolved through discussion among reviewers until consensus was reached.

The search period was set to January 1980 until October 2017. A second search was conducted to extend the search period up to 9 March 2020, i.e. before the declaration of the COVID-19 outbreak [57]). All types of studies were considered if a population’s need for healthcare was quantified and expressed in provider requirements.

Studies that exclusively focused on forecasting methods of physician supply, on inpatient care or on healthcare professionals other than physicians were excluded. Additionally, records in low- or middle-income countries were excluded, as were studies in any other language than English or German (for further details see Table 2).

**Table 2:** Selection criteria and justification

	Criterion	Justification
**Inclusion**	Quantification of need in provider requirements	Studies needed to assess a population’s need for healthcare quantitively and translate the respective need into provider requirements.
	Publication date	All studies between January 1980 and March 2020 were included in the review.
	Language	All studies available in English or German were included in the study. No other languages were included to avoid translation errors.
**Exclusion**	Forecasting supply	Studies that focused on forecasting existing supply with reference to demographic changes only and did not assess any additional need determinants were excluded.
	Inpatient care	Due to differences between inpatient and outpatient care (e.g. payment methods), studies that only focused on inpatient care were excluded.
	Non-physicians	Studies that investigated only non-physician providers (e.g. physiotherapists or nurse practitioners) were excluded because of differences regarding remuneration and data availability that could lead to biases.
	Low- and middle-income countries	Due to significant differences in healthcare resources, provision of health services and health infrastructure that are likely to influence data availability, studies estimating need for healthcare in low- or middle-income countries were excluded.

A detailed protocol including, amongst other parameters, a full list of keywords, websites, and search results can be found in the Additional file 1.

For data extraction, a framework of characteristics including the targeted physician groups and the determinants of need was designed. In a final step, the proposed criteria for estimating needs-based physician supply were added to the data extraction framework to appraise each study (see Additional file 2). The main outcomes of interest are the conceptual framework, the data basis, the model selection, the translation into physician capacity, and the integration of future trends and developments. The Mixed Methods Appraisal Tool and the appraisal tools of The Joanna Briggs Institute were consulted when defining the criteria for estimating needs-based physician supply. However, as these tools do not include methodological studies in their targeted study designs, their application was not suitable for the purpose of the methodological review [58, 59].

## Results

### Description of studies

We identified 18 articles published between 1995 and 2017 that quantify a population’s need for healthcare and further estimate needs-based supply. Most papers were published in the first decade (2010–2020) of the search period (n = 14) compared to one study in the second decade (2000–2009) and three papers between 1995 and 1999 (see Table 3).

**Table 3:** Descriptive summary of the empirical studies included in the literature review

	Frequency	Reference
**Year**	1995–1999: n = 3	[62–64]
	2005–2009: n = 1	[65]
	2010–2014: n = 8	[60, 61, 66–70]
	2015–2020: n = 6	[48, 71–75]
**Country of origin**	Australia: n = 1	[48]
	Canada: n = 2	[68, 69]
	Germany: n = 7	[60, 66, 70, 72–74, 76]
	Singapore: n = 1	[71]
	Spain: n = 1	[61]
	UK: n = 1	[67]
	USA: n = 5	[62–65, 75]
**Health systems**	Beveridge Model (single payer): n = 3	[48, 61, 67]
	Bismarck Model (multiple payer): n = 7	[60, 66, 70, 72–74, 76]
	Hybrid Model (multiple payer): n = 6	[62–65, 71, 75]
	National Health Insurance Model (single payer): n = 2	[68, 69]
**Unit of planning**	Multiple professionals: n = 9	[60, 61, 63, 66, 69, 70, 74–76]
	Eye care professionals: n = 2	[64, 71]
	Mental health professionals: n = 2	[65, 73]
	General practitioners (GPs): n = 2	[48, 67]
	Dental care: n = 1	[72]
	Oncologists: n = 1	[68]
	Otolaryngologists: n = 1	[62]

Table 2 also illustrates that most of the studies (n = 10) originated from predominantly English-speaking countries (Australia, Canada, Singapore, UK, and USA). Further, seven papers were derived from Germany and one paper from Spain. When looking at their respective health systems and market structure, most studies were conducted in multiple-payer settings (n = 13) out of which six studies were conducted in countries with hybrid health system models (USA and Singapore).

The planning unit of the included articles varied widely. Nine studies estimated needs-based supply for multiple professionals ranging from two [60] up to 43 physicians or medical specialists [61]. Papers that restricted the planning unit to one physician group targeted most frequently eye care professionals, general practitioners (GPs), and mental health professionals (n = 2, each) (see Table 3).

### Review of studies against criteria

#### Choices and reporting of the conceptual basis

##### Selection and justification of indicators of need (Criterion 1.1)

Each study included a rationale on how objective need was measured using several indicators, but the theoretical foundation differed in depth. For example, Stuckless et al. [68] selected determinants, which they assumed to influence demand for healthcare without giving further empirical verification. They stated, however, that the main indicators are based on theoretical frameworks of the Australian Medical Workforce Advisory Committee. The approach of using theoretical frameworks of other health workforce planners was also adopted by Laurence and Karnon [48] (Australia) and the Centre for Workforce Intelligence (CfWI) report (UK) [67]. Both utilized the determinants originally set out by Canadian research papers [43, 77]. Overall, using need-indicators on the basis of prior research was stated in 11 studies [48, 61–63, 67, 68, 71–75].

In contrast Lee, Jackson and Relles [64] developed an individual, multistep framework to explain the relevant domains of eye care services in their study. They first attributed diagnoses to each domain of services (problem-oriented, rehabilitative, preventive, and elective) by reviewing the ICD-9 catalogue and assigning relevant diagnoses to disease groups. Additionally, they consulted an advisory panel to review the underlying assumptions of their framework. Similarly, Albrecht et al. [66], Ansah et al. [71], Czaja et al. [60], Konrad et al. [65], Ozegowski & Sundmacher [70], Singh et al. [69] and von Stillfried & Czihal [76] developed their own frameworks for their analyses.

Empirical support for the indicators of need with respect to the chosen underlying conceptual basis was offered by five studies [60, 65, 66, 73, 74]. Albrecht et al. [73] assessed the relationship between the prevalence of psychological disorders and socioeconomic status before including the variables in their model. Albrecht et al. [66] used scientific literature and empirical studies to determine their indicators of need. They focused on morbidity measures (mortality and care dependency) and socioeconomic structure, which was hypothesized to approximate the morbidity burden of the population independent of supply. After applying factor analysis on the variables, the effect of each factor on need was approximated. Czaja et al. [60] also used factor analysis to find determinants that would explain most of the variation in the morbidity and the social structure of their study area.

Kopetsch and Maier [74] empirically tested the correlation of the German Index of Multiple Deprivation (GIMD) and need for healthcare by regressing the GIMD on utilization, morbidity and mortality before including it in their additive needs model. Konrad et al. [65] suggested using a logit regression to get the best estimate of demographic and socioeconomic factors that would predict the prevalence of serious mental illnesses, which was then used for their extrapolations.

**Fig. 2:**
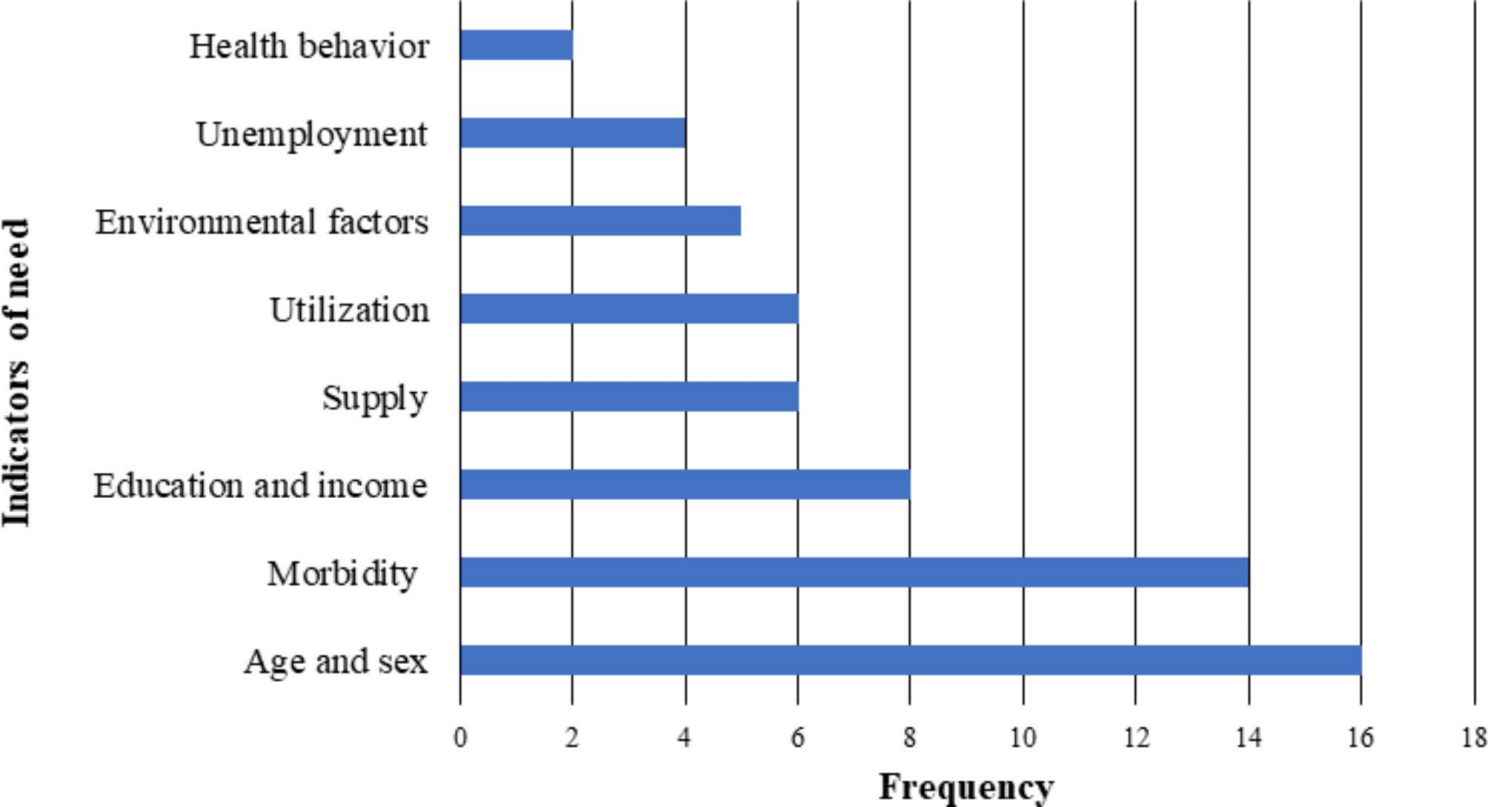
Indicators of needs-based supply employed by selected studies

Figure 2 presents the distribution of indicators of need selected in the studies. The most frequently applied exogenous determinants when quantifying the need for healthcare are the demographic variables age and sex [48, 60–67, 69–76]. Only one study [68] did not mention incorporating any of these variables. Other exogenous factors (i.e. sociodemographic status, operationalized by education and income) were included in eight studies [60, 65, 66, 71, 73, 75]. Unemployment as a measure of need was mentioned in four papers, all of them addressing the German healthcare system [60, 66, 73, 74]. Environmental factors used to operationalize the need for healthcare were employed in five studies including indicators describing regional deprivation or residency [60, 73–75] and secondhand smoke exposure as proxies [69]. Indicators of health behavior were employed in two of these papers including lifestyle risk factors (e.g. alcohol consumption, obesity) [69, 75].

Measures of morbidity were included in 14 out of 18 studies [48, 60, 62, 64–66, 69–76]. The operationalization of morbidity varied largely between incidence/prevalence rates of certain diseases [48, 64, 65, 68, 69], the dependency on care in a population [66, 73] and other morbidity groupings/disease patterns [61–63, 67, 70–72, 74, 76].

Indicators with a clear dependency on the healthcare system were divided into the categories supply and utilization, which were found in six papers each. Supply represented the current number of physicians [61, 71, 74] and productivity measures [62, 67, 68], whereas utilization was characterized by cases/visits per physician [48, 67, 70, 71, 75] and number of referrals [68]. Two studies applied both endogenous determinants in their model [67, 71].

##### Potential influence of supply (Criterion 1.2)

A key challenge of the conceptual basis concerns the potential influence of supply on need determinants. Nine papers discussed the influence of supply on some of the variables used in [61, 62, 66, 67, 69–71, 73, 75] or excluded from [66, 73] their model. For instance, Ozegowski & Sundmacher [70] acknowledged that the regional density of physicians may influence the prevalence of coded diagnoses from utilization data. Albrecht et al. [66] supported the assumption that indicators from utilization data are statistically dependent on supply (i.e. access to care), so they recommended to either apply these indicators to a limited extent or avoid them completely.

After conceptually identifying the potential influence of supply, six studies attempted to account for possible undersupply or unmet need in their estimates of physician requirements [61, 62, 67, 69, 71, 75]. For instance, Dall et al. [75] included a so-called healthcare utilization equity scenario, which models the effect of socio-demographic, economic, and geographic barriers on physician demand by comparing population groups with and without such barriers. They noted, however, that the model may still project any imbalances (under- or oversupply) into the future. Anderson et al. [62] adjusted their supply-based model by estimated numbers of uninsured people to factor in current imbalances.

Barber and López-Valcárcel [61] sought to account for current undersupply of physicians by using information about unfilled positions on the job market. Singh et al. [69] adjusted for current physician shortages using estimates on the number of people that were not attached to a general practitioner for their base-case scenario.

In contrast, Ansah et al. [71] included the estimated number of unmet care needs (underuse) in their simulation model in their ‘integrated approach’ by considering information on time differences between the date of appointment booking and the date of the patient visit (waiting lists). Similarly, the CfWI [67] used panel estimations to account for contemporary unmet need for healthcare services.

No study adjusted their model for potential overuse of medical services by patients or oversupply. Moreover, none of the studies empirically explored the correlation of supply and indicators of need.

#### Evaluating the validity of the data basis

##### External validity (Criterion 2.1)

Several data sources were used to quantify needs-based supply of physicians, which varied in their representativeness. We divided the data into four categories to systematically assess their external validity. The highest representativeness was assigned to population data for which the authors claimed that it covers the population of interest as a whole. Population data was followed by representative samples of a population and lastly, by conveniences samples. If it was not possible to assess the representativeness (i.e. the combination of several datasets whose representativeness varied and no further validity tests were conducted), we classified it as mixed data.

We found that two studies relied on population data [66, 76], one paper used information from national statistics [66] and the other employed health insurance claims of all publicly insured Germans [76]. Another two studies claimed to use representative samples for their estimations [73, 75]. By way of example, Albrecht et al. [73] applied data from an epidemiological study with information drawn from a stratified sample of the population that was considered representative on a national level. Further two data sources were classified as convenience samples [62, 70]. Anderson et al. [62] mainly used claims data without an all-payer approach (in the sense of convenience samples from some insurers). They sought to approximate the demographics of the study population as best as possible by including only the biggest health maintenance organizations in their study without any stratification.

We were unable to properly assess external validity in 18 out of 24 approaches^1^ because the authors combined various data sources [48, 60–65, 67–69, 71, 72, 74] and information on the representativeness of the data as a whole was lacking. Singh et al. [69], as one example, combined information from scientific literature and cross-sectional studies on prevalence and incidence rates without reflecting on the overall representativeness. Stuckless et al. [68] tried to ensure external validity by checking the consistency between data sets whenever possible with, however, several uncertainties on the overall representative status remaining.

Similarly, Lee, Jackson and Relles [64] declared their own representativeness of the incidence and prevalence rates as problematic in consequence of their disparate data sources.

##### Internal validity (Criterion 2.2)

Apart from the assessment of representativeness, consideration should be given to the quality and accuracy of the data, as well as to potential systematic biases and how they may be avoided. 13 studies reflected on the accuracy of their indicators and/or recognized systematic biases hampering internal validity [48, 60, 62–67, 69, 70, 73, 76]. For example, von Stillfried and Czihal [76] used health insurance claims data to estimate needs-based supply of physicians in Germany. The authors discussed potential threats to internal validity from people who changed insurers within the health system. New identification numbers would be attributed to these insurees and since the data was anonymized, would count them as two individuals.

Another general limitation of internal validity was debated due to the usage of non-repeated cross-sectional data for incidence or prevalence rates [48, 73].

The CfWI report [67], as an example of very transparent reporting, listed data quality assessments by data confidence ratings for every model parameter, differentiating confidence levels between ‘very high’, ‘high’, ‘medium’, ‘low’ and ‘n/a’ [not available]. Additionally, its authors declared assumptions concerning the data source and general data assumptions, and summarized validation approaches and remaining uncertainties [67].

Out of the 13 studies that reflected on their internal validity, six papers tried to correct for potential biases [62–64, 67, 69, 70]. Singh et al. [69], who combined information from various data sources, tried to enhance internal validity by consulting expert panels and using complementary information from the literature. Lee, Jackson and Relles [64] used a similar approach; they modified the epidemiologically derived prevalence rates used for the needs-based model with the help of an advisory panel, scientific literature or – if no data was available for certain conditions – extrapolated and rescaled prevalence-rates from utilization data.

To take potential errors based on the principles of free choice of health professionals into consideration, Ozegowski & Sundmacher [70] accounted for co-provision of care in urban regions when estimating need for healthcare.

##### Timeliness and availability (Criterion 2.3)

When looking at the timeliness of data, we found that all studies mentioned the year of collection for the main data sources. Some studies, however, stated only the year of the underlying reference publication, not the timeframe in which the data was collected [71, 72].

Among the 16 studies that reported specific years, the difference between the year of data collection and the year of workforce planning varied between 1 and 20 years. Stuckless et al. [68], the study with the largest difference, used prevalence estimates of registry data from 1989 to 2005 and survey data from 1999 to 2009 (among others) to compute the need for oncologists. Only two studies theoretically justified the utilization of different base years [70, 74]. Kopetsch and Maier [74] hypothesized that morbidity measures within one region would not change substantially over a timeframe of two years. Similarly, Ozegowski & Sundmacher [70] relied on commuters’ data, which was valid for one year after baseline under the hypothesis that commuting behavior would not vary fundamentally between consecutive years.

Terms of access to the data sources or the frequency of data reporting were not systematically reported. Only Laurence and Karnon [48] reported not only the availability of data sets used in the study but also the frequency of data collection in detail.

In general, several studies stated that they mostly relied on the latest datasets available [48, 66, 75] and that the data was collected routinely/periodically [64, 68, 70, 76].

#### Modelling and translation into physician capacity

##### Translation into provider requirements (Criterion 3.1)

After quantifying the need for healthcare, results of the empirical quantity must be related to some measure of provider requirements. Most studies employed fulltime equivalents (FTE) to translate population need for healthcare into physician capacities [48, 61–65, 67–69, 71, 75, 76]. Transformation to FTE was made by using averaged numbers of consultations, averaged minutes devoted to patientcare or predetermined working hours (e.g. 40 h weekly working time) per physician. Singh et al. [69], as one example, multiplied physician-time spent to treat a disease with the overall number of disease cases (considering yearly incidence and prevalence rates) to estimate the total service hours needed, which can be converted into FTE using the average number of hours worked per year by a physician. Likewise, Greenberg and Cultice [63] derived their FTE conversion factor for each specialty by dividing the total minutes dedicated to patient care of the base year (considering indirect and direct contact) by the number of physicians who were reportedly active.

Due to the lack of a uniform worktime recording system for physicians, Konrad et al. [65] used various sources in order to approximate the hours a physician spent in direct patient contact per year to estimate the conversion factor of needed service minutes in FTEs.

An example for translation through average consultations was provided by Dall et al. [75]. The authors used the total number of physicians of the base year to calculate the average volume of services delivered per FTE. For this purpose, they divided the yearly volume of healthcare services delivered by the base year staffing under consideration of known shortages of physicians. Similarly, von Stillfried and Czihal [76] used average health services delivered per physician (measured in so-called service points) across Germany as conversion method.

Predetermined working hours were applied by Laurence and Karnon [48] as conversion tool for FTEs. They translated the estimated hours of services needed into FTEs through 40 h weekly working time and a total number of 44 workweeks per year in order to account for potential sick/annual leave or other training absences. Lee, Jackson and Relles [64] used survey data reviewed by experts to convert services needed into actual worktime estimates. To further translate these estimates into FTE, they also used predefined working hours (48 workweeks of 42 h weekly working time).

Another approach to relate need for healthcare with healthcare supply was to adjust or contrast existing physician-to-population-ratios by morbidity measures instead of directly converting estimates of need into FTE [60, 62, 66, 70–74].

One example for contrasting need to supply was posed by Jäger et al. [72], who first translated the estimated burden of oral morbidity into required treatment time based on health insurance claims data. Subsequently, these estimates were compared to but not directly converted into physician supply (using the Gini coefficient). Likewise, Ozegowski & Sundmacher [70] related the physician headcounts with regional need for healthcare through the Concentration Index because no information on actual working hours was available to directly translate need into physician capacities.

Albrecht et al. [66] exemplified adjustment of physician-to-population ratios through need measures. First, they determined new physician-to-population ratios using the actual ratio of regions, which they estimated to have an average need for healthcare, before adjusting all regions with their self-developed need-index. In their latest work, they used the same approach but refined the composition of the need-index [73].

##### Model validation (Criterion 3.2)

Before we were able to address aspects of the model validation, we extracted the central statistical model for each study. We found three main modeling approaches in the literature review: extrapolations (also incorporating index adjustments), regression-based analyses, and simulation models (see Table 4).

**Table 4:** Summary of the criteria for operationalizing population need for healthcare for physician planning. (0/1) simplified indicates that the aspect was assessed as criterion being ‘present’ (1) or ‘not present’ (0). A full description can be found in the Additional file 2 NB: The number approaches result into n = 24 instead of n = 18 as three studies adopted several approaches

**1. Conceptual framework**	**Findings**
1.1 Selection and justification of needs indicators • Theoretical rationale (0/1) • Empirical validation (0/1)	Theoretical rationale for the indicators • n = 24 Empirical validation of indicators • n = 5
1.2 Relationship between supply and need • Potential influence (0/1) • Potential unmet need or lack of physicians (0/1) • Potential overuse or oversupply (0/1)	Discuss potential influence of supply • n = 9 Adjust potential unmet need or lack of physicians • n = 6 Adjust potential overuse or oversupply • n = 0
**2. Data basis**	**Findings**
2.1 External validity • Representativeness	Representativeness • Population data: n = 2 • Representative sample: n = 2 • Convenience samples: n = 2 • Mixed data: n = 18
2.2 Internal validity • Accuracy of indicators	Discuss accuracy of indicators • n = 14
2.3 Timeliness and availability • Survey period	Survey/recording periods (in years) • Ranges between 1–20 years
**3. Modelling and translation into physician capacity**	**Findings**
3.1 Transformation into provider requirements • Methodology	Methodology to translate estimated need into supply • FTE: n = 14 • Physician-to-population ratio adjustment: n = 10
3.2 Model selection and validation • Type of model • Justification and validation (0/1)	Type of model • Regression-based: n = 4 • Simulations: n = 9 • Extrapolations: n = 11 Validation of the model • n = 21
3.3 Level of analysis • Aggregated data (0/1) • Individual data (0/1)	Model based on aggregated data • n = 21 Model based on individual data • n = 3
**4. Integration of future trends and developments**	**Findings**
4.1 Projection variables • Selection of variables	Variables for projection models • Demographics: n = 13 • Utilization: n = 5 • Supply: n = 5 • Morbidity: n = 3 • Insurance status: n = 2 • Health behavior: n = 1
4.2 Planning horizon • Length • Validation (0/1)	Length of need projections • Ranges between 10–31 years Validation of length • n = 0

Out of these approaches, simulation models and index adjustments were found to include a theoretical justification of the model. It was generally claimed that system dynamic models, for example, were well suited for the healthcare environment, specifically for forecasting purposes, because of their adaptability and ability to model complex relationships [61, 67, 71].

Some aspects of model validation including sensitivity analyses were found in most of the studies [48, 60–68, 70–73, 75, 76]. Von Stillfried and Czihal [76], as a regression-based example, used the coefficient of determination (R^2^) to determine the model fit. They argued that although their relative risk score incorporates age and sex, which are already independent variables in the regression, the risk score still extends the amount of explained variation in the data significantly if added to the model. Likewise, Albrecht et al. [66], Jäger et al. [72], and Kopetsch and Maier [74] used R² to assess their model fit.

The HIS report from Dall et al. [75], as one example including extensive validity assessment, used a micro-simulation model, which they sought to cross-validate by comparing the predicted service-usage with alternative approaches from the literature that estimated national use of healthcare services. Additionally, they validated their methodology according to the five main types of validation recommended in the best-practice report from the International Society for Pharmacoeconomics and Outcomes Research (ISPOR).

Also, Greenberg and Cultice [63] applied cross validation and compared the findings of the extrapolation-based utilization approach with the formerly used supply-based model.

In order to validate the appropriateness of their simulation (system dynamics) models, Ansah et al. [71] consulted stakeholders and cross-checked the validity with historical data. Additionally, they conducted sensitivity analyses for every parameter used in their simulation model by varying each parameter by 25% (assuming a uniform distribution) as well as Markov chain Monte Carlo simulations.

In order to ensure the statistical stability of their model in light of the underlying disparate data sources, Lee, Jackson and Relles [64] computationally resampled (bootstrapped) the data and introduced random statistical variation through various randomization techniques.

##### Level of analysis (Criterion 3.3)

Three models identified in the literature used individual-level data to estimate needs-based supply [73, 75, 76]. Von Stillfried and Czihal [76] used service points per person on an individual level. Similarly, the IHS report employed individual-level measures of healthcare utilization (outpatient visits) [75]. In contrast, Albrecht et al. [73] used epidemiological data to estimate the correlation of socioeconomic factors and morbidity (prevalence) on an individual level before ultimately transposing these findings to regional levels.

All other models used aggregated or partially aggregated data. For instance, Stuckless et al. [68] worked with incidence rates and referral rates in their model. Ansah et al. [71] used both aggregated and partially disaggregated data such as prevalence rates of eye diseases disaggregated by demographic variables.

None of the studies thoroughly discussed the implications of using individual compared to aggregated data or vice versa. Kopetsch and Maier [74] mentioned that regional analyses may mask correlations due to averaged effects and highlight the potential of the Modifiable Areal Unit Problem when defining the level of analysis. In this context, Ozegowski & Sundmacher [70] also highlighted that smaller-scale models should be employed whenever possible.

#### Integration of future trends and developments

##### Projection variables (Criterion 4.1)

We found that 12 studies projected need for healthcare and the corresponding provider requirements into the future. Projection models were mainly based on demographic changes (population growth, migration, aging and mortality) with morbidity-levels, utilization patterns and staffing ratios assumed to remain constant [48, 61–64, 66–69, 71, 72, 75].

Indicators that were regarded as dependent on the healthcare system were applied in eight studies. Possible trends in morbidity were estimated in three studies [67–69]. For instance, Singh et al. [69] estimated future prevalence increases in each of their top ten ICD-10 diseases from 2009 to 2030, using different baseline assumptions for their scenarios. Stuckless et al. [68] suggested their morbidity trends on historical annual increases in cancer incidence rates based on cancer statistics and published evidence from the literature and assumed that these trends would continue in the future, thus holding them constant in the projection models. In contrast, the CfWI [67] tried to estimate change for healthcare need using Delphi panels.

Other variables used in projection models were insurance coverage [62, 63] and changes in health risk factors [69].

##### Planning horizon (Criterion 4.2)

The length of the projections varied between 10 years [48, 68] and 31 years [63], having a mean of 17 years. No substantive explanation (validation) was found on the length of projection. However, since projection models were mainly based on demographic changes, the length of available population projections was mentioned frequently when describing the data [61, 62, 64, 66, 67, 71, 72].

#### Overview of findings

No study was able to fully meet the guiding criteria set out in this review.

Table 4 provides an overview of the criteria used to quantify needs-based supply of physicians and the corresponding findings. Detailed information on the results of each study, also including the descriptive characteristics, can be found in the Additional files.

## Discussion

We conceptualized population need for healthcare as the latent level of avoidable or treatable morbidity in a population that is not directly measurable and requires approximation. From the conceptualization and the practical requirements of planning, we derived a set of guiding criteria that any quantitative analysis of provider requirements should consider. Complementary to previous reviews [4, 42, 78], our study focused specifically and more deeply on the relationship between need, supply, and utilization in the estimation of needs-based supply. In the following, we highlight methodological gaps along our proposed framework and suggest implications for research and policy.

Overall, needs-based physician supply was planned for a diverse portfolio of physician groups, with one up to 43 groups per study. The physician group which was targeted most frequently, either solely [48, 67] or in combination [60, 66, 69, 70, 74, 76] was GPs. One reason why there are considerable efforts to estimate GP supply presumably originates in their central role in primary care as care coordinators [79] and gate keepers [80]. They are also regarded as vital players for cost-effective provision of health services that stimulate equity in health outcomes [40]. Despite the efforts to estimate-needs based supply of GPs including projections that forecasted imbalances between need and supply [66, 67], studies failed to adequately predict the magnitude of current GP shortages [40, 81]. This highlights the importance of improving methodological approaches in areas that are identified in this review such as incorporating future trends and developments.

### Strengthening the conceptual basis and transparency of the underlying theory of need

For any need assessment, it is important to ensure transparency on the underlying conceptual basis and the development of the theoretical model as well as the chosen determinants of need.

With respect to the strategies used by the authors to theoretically justify and empirically approximate indicators of need (Criterion 1.1), it became clear from the results that these rationales were based on several, at times not fully transparent assumptions. However, a strong theoretical framework would be central to approximate latent need which cannot be measured directly. Especially when indicators of need are selected based on prior research, it is important to keep in mind that the significance of these indicators may differ over time, between health systems and even between different physician groups within a single system. Thus, the application of approaches from mostly privately funded health systems shall be considered in detail before implementing them in publicly funded systems. It remains of great importance to have a strong theoretical framework, which secures, among other things, the transferability of prior research to a new setting.

With respect to the effect of supply on estimates of need (Criterion 1.2), 14 models relied at least in parts on utilization data to infer population morbidity from coded diagnoses in different regions (14 studies) or to identify regional patterns of utilization (4 studies). No study scrutinized potential overuse of medical services by patients or oversupply in terms of the number of providers and/or services provided. The phenomenon of supply-sensitive care refers to services whose delivery depends on the density of the local supply structure, regardless of medical need for care [82, 83]. Thus, if indicators of morbidity are derived from utilization data, the needs assessment can be biased through inappropriately high or low utilization rates. For instance, Albrecht et al. [73] showed that need estimates for psychologists from utilization data was significantly lower than those derived from estimations based on epidemiological data. They argue that the gap may be explained by lower utilization rates of older adults and people with a lower socioeconomic status.

To ensure that regional differences in utilization that result from inequalities in access to care do not bias estimates of need, Sundmacher et al. [44] recommend an adjustment for expected utilization at the level of the respective planning area. Conceptually, the expected utilization should be based on exogenous factors that are well correlated with regional utilization (such as age and sex), so that system effects associated with supply can be excluded. However, it is also conceivable that population need for healthcare, which is identified following the adjustment, does not lead to actual service utilization, if there is no perceived need.

Altogether, implications arising from an adjustment for unmet need or utilization pattern require careful deliberation and design. Standardised methods to systematically account for overutilization and/or oversupply in order to redress resulting inequalities in access to healthcare are yet to be developed.

### Strengthening the availability and validity of the data basis

The data basis should allow for coverage of the entire population or at least a representative sample on small area level for which services and providers are to be planned and should adequately reflect the indicators being measured. The smaller the geographic area for which providers are to be planned, the more challenging it will be to secure appropriate data to fulfill these criteria.

In terms of external validity (Criterion 2.1), Konrad et al. [65] remark that national census data, which is most frequently used in the records reviewed in this study – despite being a source that is independent of healthcare supply and utilization – might also be subject to systematic errors (e.g. incorrectly assessing people with lower socioeconomic status). Caution is also advised when relying on claims data of single health insurance providers as noted by Ozegowski & Sundmacher [70]. The characteristics of the population regarding socioeconomic status, age and sex may differ among the health insurance providers and thus may not be representative for the entire population. In addition, the main purpose of the data remains billing health services used by patients. Therefore, the data in its origins depends on the billed services among regions. Thus, one should always consider healthcare system-dependent aspects when relying on utilization data (e.g. regional (deprivation) and socioeconomic inequalities in access to primary care), which in turn influence the overall representativeness of the population and ultimately lead to errors in the results if not being accounted for in the analysis.

Measuring internal validity (Criterion 2.2) was not a straightforward task. Eleven approaches were found to discuss the accuracy of their indicators in accordance with our definition. Vital information on the data collection (year, methodology or limitations) and other quality measures were not transparently reported. Aspects which might hamper internal validity such as coding accuracy when using utilization data [49, 50], were not consistently disclosed. Future studies should transparently review data quality, and, if necessary, outline and discuss potential inaccuracies or biases.

Another neglected area was reflecting on timeliness of the data when estimating needs-based supply of physicians (Criterion 2.3). Most commonly, the year of collection was stated without discussing further implications. Potential limitations arising, for instance, from the usage of datasets from different years in the same model were not recognized. It should be acknowledged that great variation in source years influence the robustness of the findings, specifically if the variables are expected to change substantially over time [75]. Thus, future studies need to clearly establish the appropriateness of using different source years, if data cannot be collected in similar timeframes, either through evidence from literature or expert opinions to reduce this potential source of errors.

The main reason to challenge validity of the data basis was attributed to the lack of suitable data. [65, 70] pointed out specifically that based on their theoretical rationale and conceptual relationship with the chosen concept of need, they would have preferred to include additional variables, but were unable to do so due to restrictions in data availability. The lack of appropriate epidemiological data was further highlighted by two papers [65, 71]. Additional information on consultation lengths and morbidity levels as well as physician productivity and working hours in direct patient contact, which were found to be missing the most, would be necessary to ensure more precise estimates of needs-based physician-supply.

### Improved modelling and translation into physician capacity

The selection of appropriate statistical models depends on the characteristics of the data and needs to be reasonably justified. Sensitivity analyses and model validation testing should be standard procedure to evaluate the selected model. A special focus should lie on the level of analysis to foresee ecological fallacies. The more complex the model, the more carefully it should be described to ensure replicability.

The translation into physician capacity (Criterion 3.1) requires highly sensitive assumptions [42, 77] about productivity (i.e. units of service per hour of work and time required for a service). Despite the fact that Delphi panels were consulted in some cases to approximate the duration of patient visits, comprehensive surveys to measure physician time in direct contact with patients were lagging behind. Moreover, few studies attempted to gauge the nature and direction of potential bias that originated from the usage of averages minutes per FTE and other surrogates as translation factor. This seems like a missed opportunity. So, methods to handle uncertainty, which originated from missing or low-quality data, merit adoption in future studies.

Model selection and subsequent validation are the two main aspects for establishing confidence and trust in the model chosen for estimating needs-based supply (Criterion 3.2). Nevertheless, only studies that used System Dynamic models or index adjustments theoretically justified their statistical model. Although some forms of validation were found in a large proportion of studies, systematically assessing the model’s accuracy was found in but a few [64, 71, 75]. Moreover, transparent reporting of the purpose of the model and how the model is fitting in the setting was overall neglected. Guidelines such as the ISPOR report on Model Transparency and Validation [54] could offer assistance for the technical and non-technical documentation of the model as well as for applying validation tests.

The level of analysis is another central yet neglected aspect of the feasibility criterion (Criterion 3.3). Some publications have related variables of utilization to (small-scale) exogenous factors and/or classified morbidity groups in order to approximate population need for healthcare. The potential of ecological fallacies in aggregated models, however, should have been further assessed and discussed. For planning purposes, it is important to consider that individual data may yield more robust results than aggregated models [44]. Nevertheless, the level of analysis is highly dependent on the quality and availability of data and thus, not always influenceable by the authors.

### Incorporating future trends and developments

Studies estimating needs-based supply should not only gauge the current level of supply needed, but also incorporate future trends and developments in order to make the findings suitable for application to workforce planning.

The impact of demographic changes (Criterion 4.1) was mostly accounted for by changes in age and sex structures in the population. However, when the effect of age on health changes over time (for instance if, on average, 65-year-olds can expect to be healthier and hence have less healthcare needs than 65-year-olds 20 years ago), age-based projection models may generate misleading estimates of future resource requirements. For example, Stephan et al. [84] showed that there are differences in health statuses of older adults depending on the period they were born in, specifically if an economic or political crisis had occurred in their early life. In future, the COVID-19 pandemic might also influence the morbidity pattern based on age and sex as females, for example, were found to be of higher risk to develop a post-COVID-19 syndrome [85, 86]. Several approaches exist to test and, if required, relax the assumption of a fixed relationship over time between indicators such as age and health status [33], and merit adoption in future research.

Similarly, the average service output per provider (productivity) may vary over time [52]. The CfWI [67] suggested that workload of - in their case - general practitioners had changed over the years and were likely to continue to change in the future. The average rate of service delivery per physician will depend on the availability and use of other resources. New models of service delivery, aimed at increasing the productivity of resources (i.e. more output from a given level or combination of resources), may therefore change the human resources required to meet the needs of a population. Also, the income of physicians was found to correlate with productivity levels, leading to a decline in productivity if compensation for physicians was set over the target income [87]. Thus, it is important to track the changes in service delivery to be able to incorporate them for future predictions, acknowledging, however, that some uncertainty in the findings will remain.

When looking at the predictions of the studies in our review, three out of thirteen studies that projected need for healthcare into the future integrated trends in morbidity. The implications of rising morbidity levels were mostly neglected. Higher rates of chronic diseases and multimorbidity might influence the duration of physician visits [88] as well as the utilization rates [89–91] specifically for older adults. However, the main challenge in this respect lies in the accurate prediction of changes in morbidity over time, which is not only dependent on an adequate dataset but also on the temporal stability of morbidity trends. Additional studies are needed to improve the robustness of prediction of trends in morbidity.

Planning horizons (Criterion 4.2) varied in our review from 10 to 31 years, with no substantive underlying assumptions. Mostly, prediction lengths were dependent on the availability of population forecasts. Van Greuningen, Batenburg and Van der Velden [51] suggested in the context of GP projections that shorter periods (i.e. 5 years) yield a higher accuracy compared to longer predictions (10–15 years). Albrecht et al. [73] as well as Dall et al. [75] further argued that both healthcare system and need indicators are fast-changing, which do not allow for long-term predictions and require frequent updates. Similarly, Stuckless et al. [68] suggested annual recalculation of the models. However, short-term predictions need to be traded off against the duration of physician licenses, which may last for 20–30 years and other influencing factors such as low predicted numbers of physicians on the number of medical residencies. Thus, it would be recommended to create guidelines on the planning horizon and the frequency of updates, which are required to ensure basic robustness of the predictions and, consequently, to avoid high levels of future over- or undersupply of physicians.

Unforeseen events, such as the outbreak of the COVID-19 pandemic in March 2020 are challenging to predict [92] and thus, difficult to incorporate into the general workforce planning. Although efforts have been made to elaborate different scenarios of future pandemics and epidemics [93], it is still very challenging to predict which scenario will occur. However, these scenarios in combination with findings from past pandemics can be used to identify potential gaps in current health workforce planning. For example, calculating service targets of healthcare professionals required in case of a pandemic, exemplified for the influenza [94] and COVID-19 pandemic [95], can be applied as a tool to complement current workforce planning approaches and identify potential shortages in healthcare supply. Yet, additional research is needed to test whether these approaches accurately predict the workforce supply needed in a population.

While efficient health workforce planning that incorporate future trends in healthcare needs constitutes the basis for meeting a population’s need for healthcare, improved preparedness plans including estimations of health workforce providers needed in cases of a pandemic in addition to methods for rapidly expand available supply [96], are central resource to effectively respond to emerging care needs in times of crisis [93, 96].

### Strengths and limitations of this review

In contrast to previous reviews [4, 42], we focused on the methodological approaches used to assess the need for healthcare in a population and used to translate need into physician requirements in order to identify current gaps in workforce planning, which can be addressed by workforce planners or policy makers in future estimations. Compared to the most recent review [78], we consider estimations for all outpatient physicians irrespective their specialty and follow a clear framework to synthesize the results.

To extend our findings of English-language studies, with their resulting emphasis on English-speaking countries, we also include German-language studies. This is relevant since in Germany in particular, recent healthcare reforms have emphasized the importance of morbidity-oriented planning, which is reflected in growth of potentially relevant studies. Nevertheless, studies in other languages were not considered to avoid translation errors, which constitutes a limitation to this review.

In addition to including studies in German, we hand-searched the websites of leading national institutions (worldwide) concerned with health workforce planning [see Additional file 1] in an attempt to reduce location bias. However, language restrictions might have influenced the studies found on national and local websites.

One limitation of the review might be seen in the fact that we did not use validated critical appraisal tools to assess the quality of our studies. Instead, we measured the quality of the studies indirectly through the criteria for estimating needs-based supply, which encompass many aspects of common appraisal tools (e.g. the Critical Appraisal Skills Programme (CASP) of the Oxford Centre for Triple Value Healthcare [97]) but in a format specifically fitted to our objective.

The scope of our review included the perspective of need estimations and its peculiarities, only. As a next step it would be important to look at supply-side modelling to complement our findings. Important aspects beyond the scope of estimated inflows and outflows such as regional distribution of physicians (access to care), the constellation of the workforce (female doctors, medical emi- and immigration) and changes in work-life balance as well as staff satisfaction should be addressed [67, 70, 98]. Moreover, long- and short-term strategies to maintain and increase physician supply to secure sufficient capacities to meet a population’s need for healthcare – including exceptional situations such as of a pandemic or a natural disaster – need to be in place [99]. In this respect, options for the delivery of health services through a multidisciplinary health workforce team [100] and the potential of telemedicine should also be realized [101].

## Conclusions

We reviewed methodological approaches to quantify appropriate physician supply with reference to population needs. The list of criteria set out in this study serves as a transparent guide for operationalizing the latent construct of needs-based supply with respect to critical challenges. The review targets not only health services researchers but also policymakers in the field of health workforce capacity planning to support future estimates.

Our criteria-led appraisal of the studies shows distinct heterogeneity in the model approaches, data basis and processing, complexity, and significance of current international studies on needs-based supply of physicians. As none of the studies fully meet the guiding criteria, possibilities for methodological improvements were identified across the studies. Some approaches have distinct strengths (e.g. extensive model validation) combined with weaknesses (e.g. modelling imbalances of supply and demand into the future). Thus, we detect areas where there is insufficient reporting in the result section and offer suggestions on how to improve the accuracy of estimating needs-based supply in the discussion.

Quantifying population need for healthcare and translating it into provider capacities remains a complex challenge. Decisions related to health workforce capacity planning should be made carefully with regard to the selection and quantification of the required indicators, the choice of database and the modelling approach, while also taking into account future developments.

## Electronic supplementary material

Below is the link to the electronic supplementary material.

Additional file 1: Review Protocol. Supplementary Table 1: Reporting Search History

Additional file 2: Overview of the criteria for operationalizing population need for healthcare for physician planning

## Data Availability

A summary of all studies used for this systematic review can be found in the Additional material. All studies used for this review can be accessed online.

## Footnotes

1 *The number of approaches (n = 24) exceeds the number of studies (n = 18) because three studies adopted several approaches.*
