# Supplementary material for: Assessing needs-based supply of physicians: a criteria-led methodological review of international studies in high-resource settings
Source: BMC Health Serv Res. 2023 May 31;23:564. doi: 10.1186/s12913-023-09461-0 (PMC10231959; doi:10.1186/s12913-023-09461-0)
Supplement: Supplementary file 1 — Additional file 1: Review Protocol. Supplementary Table 1: Reporting Search History [file 12913_2023_9461_MOESM1_ESM.pdf]

Additional file 1: Review Protocol[1]

Summary	Assessing needs-based supply of physicians: A criteria-led methodological review of international studies in high-resource settings
Aim	The aim is to apply a set of criteria to review methodological approaches to estimate needs-based physician requirements.
Inclusion criteria	• Empirical studies<br>• Publication dates: starting January 1980<br>• Language: English, German
Exclusion criteria	• No quantification of need<br>• Forecasting supply<br>• Inpatient care<br>• Non-physicians<br>• Language restrictions<br>• Low- and middle-income countries
Information sources	Scientific database:<br>• Social, life and health sciences: Web of Science Core Collection, Science Direct<br>• Biomedical: PubMed<br>Grey literature: complementary search in OpenGrey<br>Searches on predefined, international websites (full list including links, see 2.2)<br>Hand search of relevant sources in published studies and author searches
Selection process	The review process was conducted by two independent reviewers; disagreements were resolved through discussion until consensus was reached.

[1] Adapted from Shamseer L, Moher D, Clarke M, Ghersi D, Liberati A, Petticrew M, Shekelle P, Stewart L, PRISMA-P Group. Preferred reporting items for systematic review and meta-analysis protocols (PRISMA-P) 2015: elaboration and explanation. BMJ. 2015 Jan 2;349 (jan02 1):g7647.

Supplementary Table 1: Reporting Search History

1.1. Electronic Database

**Database, provider**		**Web of Science Core Collection, Thomson Reuters**	**Update**
Search date (dd/mm/yyyy)		05/04/2017	09/03/2020
Period or update status		1963-05/04/2017	05/04/2017-09/03/2020
#	Search	Results	Results
1	TOPIC: ("workforce planning")	680	/
2	TOPIC: ("capacity planning")	2,024	/
3	TOPIC: ("human resource*")	27,379	/
4	TOPIC: ("planning")	343,331	/
5	TOPIC: ("service requirement*")	2,857	/
6	TOPIC: ("health")	1,545,733	/
7	TOPIC: ("physician*")	252,153	/
8	#7 AND #6	80,602	/
9	#8 AND #5	8	/
10	#8 AND #1	66	/
11	#8 AND #2	7	/
12	#8 AND #4 AND #3	70	/
14	#13 OR #12 OR #11 OR #10 OR #9	**132**	**91**

**Database, provider**		**PubMed, U.S. National Library of Medicine**	**Update**
Search date (dd/mm/yyyy)		16/05/2017	09/03/2020
Period or update status		1980-16/05/2017	16/05/2017-09/03/2020
#	Search	Results	Results
1	TOPIC: ("need")	391,069	/
2	TOPIC: ("capacity planning")	83	/
3	TOPIC: ("health human resource")	104	/
4	TOPIC: ("demand")	67427	/
5	TOPIC: ("healthcare")	166,724	/
6	TOPIC: ("workforce planning")	511	/
7	TOPIC: ("forecast") / ("projection")	4,400 / 48,535	/
8	#6 AND #5	85	/
9	#6 AND #5 AND #1	25	/
10	#6 AND #5 AND #4	24	/
11	#6 AND #5 AND #7	2	/
12	#6 AND #3	4	/
13	#6 AND #7	8	/
14	#3 AND #1	25	/
15	#3 AND #4	13	/
16	#9 OR #10 OR #11 OR #12 #13 OR #14 OR #15	**186**	**3**

**Database, provider**		**Science Direct, Elsevier**	**Update**
Search date (dd/mm/yyyy)		20/09/2017	09/03/2020
Period or update status		1980-20/09/2017	20/09/2017-09/03/2020
#	Search	Results	Results
1	TOPIC: ("need")	6,016,181	/
2	TOPIC: ("capacity planning")	5,326	/
3	TOPIC: ("health human resource")	403	/
4	TOPIC: ("demand")	1,537,913	/
5	TOPIC: ("healthcare")	392,618	/
6	TOPIC: ("workforce planning")	1,344	/
7	TOPIC: ("forecast") / ("projection")	244,797 / 698,167	/
8	#6 AND #3	26	/
9	#6 AND #5 AND #4 AND #1	298	/
10	#6 AND #7 AND #5 AND #4 AND #1	65	/
11	#6 AND #7 AND #3	10	/
12	#2 AND 5 AND #7	117	/
13	#8 OR #9 OR #10 OR #11 OR #12	**516**	**187**

Summary: full search history

**Database, provider**	**Search date (dd/mm/yyyy)**	**Period or update status**	**Results**	**Update**
Web of Science Core Collection, Thomson Reuters	04/04/2017	1963 – 05/04/2017	132	91
PubMed, U/S/ National Library of Medicine	16/05/2017	1980 – 16/05/2017	186	3
Science Direct, Elsevier	16/05/2017	1980 – 20/09/2017	516	187
Overall including duplicats			834	281
Overall no duplicats			**790**	**203**

1.2. List of websites included in the identification process[2]

International:

- Andean network of Observatories for human resources for health https://www.emro.who.int/entity/health-workforce/index.html
- European Commission on Public Health, health workforce http://ec/europa/eu/health/workforce/policy/index_en/htm
- European Observatory on Health Systems and Policies http://www/euro/who/int/en/about-us/partners/observatory
- GHWA https://www.who.int/teams/health-workforce/network
- Health Cluster EU http://healthclusternet/eu
- HRH Global Resource Centre http://www/hrhresourcecenter/org/
- Joint Action on Health Workforce Planning and Forecasting http://www/euhwforce/eu/
- KIT http://www/kit/nl/kit/en/
- The Health Systems and Policy Monitor
    - http://www/hspm/org/mainpage/aspx
- OECD http://www/oecd/org/
- World Bank http://www/worldbank/org/
- WHO Health Workforce https://www.who.int/health-topics/health-workforce
- WHO EURO Health Evidence Network (HEN)
    - http://www/euro/who/int/en/data-and-evidence/evidence-informed-policy-making/health-evidence-network-hen
- WHO Collaborating Centers focusing on HRH
    - University of Western Cape http://www/uwc/ac/za/Faculties/CHS/soph/Pages/WHO-Collaborating-Center-/aspx
    - University of Illinois at Rockford http://ncrhp/uic/edu/index/cfm?id=1031&b=1003&page=World%20Health%20Organization%20%28WHO%29%20Collaborating%20Centre
    - McMaster University http://nursing/mcmaster/ca/WHO_collaborating_centre/html
    - WHO Collaborating Center on Health Workforce Policy and Planning - http://whoccworkforce/ihmt/unl/pt/

Country specific:

- Canada
    - Health Canada (human resources strategy) http://www/hc-sc/gc/ca/hcs-sss/hhr-rhs/strateg/index-eng/php
    - CHHRN http://www/hhr-rhs/ca/
- Netherlands
    - NIVEL http://www/nivel/nl/en
    - Advisory Committee on Medical Manpower Planning http://www/capaciteitsorgaan/nl/Publicaties/tabid/68/language/en-US/Default/aspx
- Austria
    - Gesundheitsportal https://www/gesundheit/gv/at/Portal/Node/ghp/public
    - Hauptverband der österreichischen Sozialversicherungsträger http://www/hauptverband/at/portal27/hvbportal/content?contentid=10007/752050&viewmode=content
- Sweden
    - Ministry of Health and Social Affairs http://www/government/se/sb/d/2061
    - National Board of Health and Welfare http://www/socialstyrelsen/se/english

[2] Adapted from Tomblin Murphy, G., Birch, S., MacKenzie, A. *et al.* A synthesis of recent analyses of human resources for health requirements and labour market dynamics in high-income OECD countries. *Hum Resour Health* **14**, 59 (2016). https://doi.org/10.1186/s12960-016-0155-2

- UK
    - Centre for Workforce Intelligence http://www/cfwi/org/uk/
    - Department of Health https://www/gov/uk/government/publications?departments[]=department-of-health
    - Health Education England https://hee/nhs/uk/
- Germany
    - IGES Institut GmbH https://www/iges/com/
    - Zentralinstitut für die kassenärztliche Versorgung http://www/zi/de/
    - GKV Spitzenverband https://www/gkv-spitzenverband/de/
    - Wissenschaftliches Institut für Gesundheitsökonomie und Gesundheitssystemforschung http://www/wig2/de/
